# Draft Genome Sequences of 24 *Lactococcus lactis* Strains

**DOI:** 10.1128/genomeA.01737-16

**Published:** 2017-03-30

**Authors:** Lennart Backus, Michiel Wels, Jos Boekhorst, Annereinou R. Dijkstra, Marke Beerthuyzen, William J. Kelly, Roland J. Siezen, Sacha A. F. T. van Hijum, Herwig Bachmann

**Affiliations:** a Centre for Molecular and Biomolecular Informatics, Radboud Institute for Molecular Life Sciences, Radboud umc, Nijmegen, the Netherlands; b TI Food and Nutrition, Wageningen, the Netherlands; c NIZO food research B.V., Kluyver Centre for Genomics of Industrial Fermentations, Ede, the Netherlands; d Grasslands Research Centre, AgResearch Limited, Palmerston North, New Zealand; e Microbial Bioinformatics, Ede, the Netherlands; f Systems Bioinformatics, Faculty of Earth and Life Sciences, VU University Amsterdam, Amsterdam, the Netherlands

## Abstract

The lactic acid bacterium *Lactococcus lactis* is widely used for the production of fermented dairy products. Here, we present the draft genome sequences of 24 *L. lactis* strains isolated from different environments and geographic locations.

## GENOME ANNOUNCEMENT

*Lactococcus lactis* is a Gram-positive bacterium that is predominantly found on plant material and in the dairy environment (1, 2). It is extensively used in dairy fermentations (3), which is mainly due to its role in the development of texture and flavor through, e.g., proteolysis and the production of volatile flavor compounds (4). It also contributes to food preservation through the production of organic acids and bacteriocins such as nisin (5). Four *L. lactis* subspecies have been defined (6): subsp. *lactis* (7), subsp. *cremoris* (8), subsp. *hordniae* (9), and subsp. *tructae* (10). In this study we report the draft genome sequences of 24 *L. lactis* strains, of which 23 belong to subspecies *lactis* and one (LMG8520) is the type strain for the subspecies *hordniae* (Table 1) (11). However, we found in a detailed phylogenetic and comparative genome analysis that strain LMG8520 has a *L. lactis* subsp. *lactis* genotype.

The strains were grown overnight in 5 mL LM17 broth at 30°C. After propagation in fresh medium, cells were harvested during midexponential growth and total DNA was isolated as previously described (11) with the following modifications. Cell pellets were resuspended in a buffer [6.7% sucrose, 1 mM EDTA, 50 Mm TrisHCl (pH 8.0)] and incubated with RNase (0.5 mg/mL) and lysozyme (2 mg/mL) at 37°C for 1 h. Subsequently, cells were lysed by treating the samples with SDS (1% wt/vol final concentration) at 37°C for 10 min. The total DNA was extracted with phenol-chloroform, precipitated with isopropanol and sodium acetate (12), and dissolved in sterile water.

Whole-genome sequencing was performed at GATC Biotech (Konstanz, Germany) with 50 bp paired-end libraries on an Illumina HiSeq 2000. Raw sequence reads of each of the genomes were assembled *de novo* using IDBA (13) with default parameters at a target coverage of 50×. This resulted in draft genomic sequences for 24 *L. lactis* strains (Table 1). Annotation of the contig sequences was performed by the RAST server (14).

**TABLE 1:** Overview of the 24 *L. lactis* strains in NCBI BioProject PRJNA294255

Strain	Original strain name or culture collection no.	Subspecies	Accession no.	Source of isolation	Country of isolation
ATCC 19435^*a*^	OJ	*lactis*	LKLC00000000	Milk (dairy starter) (11)	Denmark
DRA4		*lactis* biovar *diacetylactis*	LIWD00000000	Dairy starter (11)	the Netherlands
E34		*lactis*	LKLD00000000	Silage (11)	the Netherlands
K231		*lactis*	LKLE00000000	White kimchi (11)	Japan
K337		*lactis*	LKLF00000000	White kimchi (11)	Japan
KF134		*lactis*	LKLJ00000000	Alfalfa and radish (15)	New Zealand
KF146		*lactis*	LKLK00000000	Alfalfa and radish (15)	New Zealand
KF196		*lactis*	LKLL00000000	Japanese kaiware shoots (15)	New Zealand
KF201		*lactis*	LKLM00000000	Sliced mixed vegetables (15)	New Zealand
KF24		*lactis*	LKLH00000000	Alfalfa sprouts (15)	New Zealand
KF282		*lactis*	LKLN00000000	Mustard and cress (15)	New Zealand
KF67		*lactis*	LKLI00000000	Grapefruit juice (15)	New Zealand
KF7		*lactis*	LKLG00000000	Alfalfa sprouts (15)	New Zealand
Li-1		*lactis*	LKLO00000000	Grass (11)	Belgium
LMG14418	TM 147	*lactis*	LKLT00000000	Bovine milk (11)	Belgium
LMG8520^*a*^	HC-1-1	*hordniae*	LKLP00000000	Leaf hopper (11)	United States
LMG8526	B 6113	*lactis*	LKLQ00000000	Chinese radish (11)	United Kingdom
LMG9446	21L	*lactis*	LKLR00000000	Frozen peas (11)	United Kingdom
LMG9447^*b*^	31L	*lactis*	LKLS00000000	Frozen peas (11)	United Kingdom
M20		*lactis* biovar *diacetylactis*	LKLU00000000	Soil (11)	the Netherlands
ML8	NCDO1994	*lactis*	LKLV00000000	Dairy starter (11)	United Kingdom
N42		*lactis*	LKLW00000000	Soil and grass (11)	the Netherlands
NCDO895	X62	*lactis*	LKLX00000000	Dairy starter (11)	United Kingdom
UC317		*lactis*	LKLY00000000	Dairy starter (11)	Ireland

^*a*^Type strain.

^*b*^This strain was incorrectly labeled as LMG9449 in references 11 and 16.

### Accession number(s).

The genome sequences of the 24 *L. lactis* strains have been deposited as whole-genome shotgun projects at DDBJ/EMBL/GenBank under the accession numbers listed in Table 1.

## References

[1] Van Hylckama Vlieg JET, Rademaker JLW, Bachmann H, Molenaar D, Kelly WJ, Siezen RJ 2006 Natural diversity and adaptive responses of *Lactococcus lactis*. Curr Opin Biotechnol 17:183–190. doi:10.1016/j.copbio.2006.02.007. 16517150

[2] Kelly WJ, Ward LJH, Leahy SC 2010 Chromosomal diversity in *Lactococcus lactis* and the origin of dairy starter cultures. Genome Biol Evol 2:729–744. doi:10.1093/gbe/evq056. 20847124 PMC2962554

[3] Siezen RJ, Renckens B, van Swam I, Peters S, van Kranenburg R, Kleerebezem M, de Vos WM 2005 Complete sequences of four plasmids of *Lactococcus lactis* subsp. *cremoris* SK11 reveal extensive adaptation to the dairy environment. Appl Environ Microbiol 71:8371–8382. doi:10.1128/AEM.71.12.8371-8382.2005. 16332824 PMC1317451

[4] Smit G, Smit BA, Engels WJM 2005 Flavour formation by lactic acid bacteria and biochemical flavour profiling of cheese products. FEMS Microbiol Rev 29:591–610. doi:10.1016/j.femsre.2005.04.002. 15935512

[5] Cotter PD, Hill C, Ross RP 2005 Bacteriocins: developing innate immunity for food. Nat Rev Microbiol 3:777–788. doi:10.1038/nrmicro1273. 16205711

[6] Schleifer KH, Kraus J, Dvorak C, Kilpper-Bälz R, Collins MD, Fischer W 1985 Transfer of *Streptococcus lactis* and related streptococci to the genus *Lactococcus* gen. nov. Syst Appl Microbiol 6:183–195. doi:10.1016/S0723-2020(85)80052-7.

[7] Lister JB 1873 A further contribution to the natural history of bacteria: and the germ theory of fermentative changes. Q J Microsc Sci 13:380–408.

[8] Orla-Jensen S 1919 The lactic acid bacteria. Host & Sons, Co., Copenhagen, Denmark.

[9] Latorre-Guzman BA, Kado CI, Kunkee RE 1977 *Lactobacillus hordniae*, a new species from the leafhopper (*Hordnia circellata*). Int J Syst Bacteriol 27:362–370. doi:10.1099/00207713-27-4-362.

[10] Pérez T, Balcázar JL, Peix A, Valverde A, Velázquez E, de Blas I, Ruiz-Zarzuela I 2011 *Lactococcus lactis* subsp. *tructae* subsp. nov. isolated from the intestinal mucus of brown trout (*Salmo trutta*) and rainbow trout (*Oncorhynchus mykiss*). Int J Syst Evol Microbiol 61:1894–1898. doi:10.1099/ijs.0.023945-0. 20833888

[11] Siezen RJ, Bayjanov JR, Felis GE, van der Sijde MR, Starrenburg M, Molenaar D, Wels M, van Hijum SAFT, van Hylckama Vlieg JE 2011 Genome-scale diversity and niche adaptation analysis of *Lactococcus lactis* by comparative genome hybridization using multi-strain arrays. Microb Biotechnol 4:383–402. doi:10.1111/j.1751-7915.2011.00247.x. 21338475 PMC3818997

[12] Sambrook J, Russell DW 2001 Molecular cloning, Vols. 1, 2, 3 Cold Spring Harbor Laboratory Press, Cold Spring Harbor, NY.

[13] Peng Y, Leung HC, Yiu SM, Chin FY 2012 IDBA-UD: a *de novo* assembler for single-cell and metagenomic sequencing data with highly uneven depth. Bioinformatics 28:1420–1428. doi:10.1093/bioinformatics/bts174. 22495754

[14] Aziz RK, Bartels D, Best AA, DeJongh M, Disz T, Edwards RA, Formsma K, Gerdes S, Glass EM, Kubal M, Meyer F, Olsen GJ, Olson R, Osterman AL, Overbeek RA, McNeil LK, Paarmann D, Paczian T, Parrello B, Pusch GD, Reich C, Stevens R, Vassieva O, Vonstein V, Wilke A, Zagnitko O 2008 The RAST Server: Rapid Annotations using Subsystems Technology. BMC Genomics 9:75. doi:10.1186/1471-2164-9-75. 18261238 PMC2265698

[15] Kelly WJ, Davey GP, Ward LJ 1998 Characterization of lactococci isolated from minimally processed fresh fruit and vegetables. Int J Food Microbiol 45:85–92. doi:10.1016/S0168-1605(98)00135-4. 9924939

[16] Rademaker JLW, Herbet H, Starrenburg MJC, Naser SM, Gevers D, Kelly WJ, Hugenholtz J, Swings J, van Hylckama Vlieg JET 2007 Diversity analysis of dairy and nondairy *Lactococcus lactis* isolates, using a novel multilocus sequence analysis scheme and (GTG)5-PCR fingerprinting. Appl Environ Microbiol 73:7128–7137. doi:10.1128/AEM.01017-07. 17890345 PMC2168189

